# The clonogenic assay: robustness of plating efficiency-based analysis is strongly compromised by cellular cooperation

**DOI:** 10.1186/s13014-020-01697-y

**Published:** 2020-10-29

**Authors:** Nikko Brix, Daniel Samaga, Roman Hennel, Katharina Gehr, Horst Zitzelsberger, Kirsten Lauber

**Affiliations:** 1 Department of Radiation Oncology, University Hospital, LMU München, Marchioninistrasse 15, 81377 Munich, Germany; 2 Research Unit Radiation Cytogenetics, Helmholtz Center Munich, German Research Center for Environmental Health GmbH, Neuherberg, Germany; 3 Clinical Cooperation Group ‘Personalized Radiotherapy in Head and Neck Cancer’ Helmholtz Center Munich, German Research Center for Environmental Health GmbH, Neuherberg, Germany; 4 German Cancer Consortium (DKTK), Munich, Germany

**Keywords:** Clonogenic assay, Colony formation assay, Reproductive survival, Plating efficiency, Cellular cooperation

## Abstract

**Background:**

The clonogenic assay is a versatile and frequently used tool to quantify reproductive cell survival in vitro. Current state-of-the-art analysis relies on plating efficiency-based calculations which assume a linear correlation between the number of cells seeded and the number of colonies counted. The present study was designed to test the validity of this assumption and to evaluate the robustness of clonogenic survival results obtained.

**Methods:**

A panel of 50 established cancer cell lines was used for comprehensive evaluation of the clonogenic assay procedure and data analysis. We assessed the performance of plating efficiency-based calculations and examined the influence of critical experimental parameters, such as cell density seeded, assay volume, incubation time, as well as the cell line-intrinsic factor of cellular cooperation by auto-/paracrine stimulation. Our findings were integrated into a novel mathematical approach for the analysis of clonogenic survival data.

**Results:**

For various cell lines, clonogenic growth behavior failed to be adequately described by a constant plating efficiency, since the density of cells seeded severely influenced the extent and the dynamics of clonogenic growth. This strongly impaired the robustness of survival calculations obtained by the current state-of-the-art method using plating efficiency-based normalization. A novel mathematical approach utilizing power regression and interpolation of matched colony numbers at different irradiation doses applied to the same dataset substantially reduced the impact of cell density on survival results. Cellular cooperation was observed to be responsible for the non-linear clonogenic growth behavior of a relevant number of cell lines and the impairment of survival calculations. With 28/50 cell lines of different tumor entities showing moderate to high degrees of cellular cooperation, this phenomenon was found to be unexpectedly common.

**Conclusions:**

Our study reveals that plating efficiency-based analysis of clonogenic survival data is profoundly compromised by cellular cooperation resulting in strongly underestimated assay-intrinsic errors in a relevant proportion of established cancer cell lines. This severely questions the use of plating efficiency-based calculations in studies aiming to achieve more than semiquantitative results. The novel approach presented here accounts for the phenomenon of cellular cooperation and allows the extraction of clonogenic survival results with clearly improved robustness.

## Background

The clonogenic assay is widely used to test reproductive cell survival in vitro [1]. Developed already in the 1950s by Puck and Marcus [2], it has proven a powerful methodology to assess sensitivity towards radiotherapy, chemotherapy, as well as molecularly targeted therapy, and undoubtedly represents the in vitro gold standard in this regard [3–10]. The mathematical analysis of clonogenic survival data relies on the major premise that under untreated conditions the relation between the number of seeded cells and the number of resulting colonies is a linear one approximated by a constant for normalization: the plating efficiency (PE).

However, the complexity of in vitro cell proliferation which is influenced by several parameters including auto- and paracrine mechanisms [11] questions whether clonogenic growth can be adequately assessed by the current state-of-the-art analysis for a broad range of cell types. Here, we therefore examined the impact of parameters, such as cell density seeded, assay volume, incubation time, and cellular cooperation by auto-/paracrine growth stimulation, on the clonogenic assay with particular focus on the robustness of the survival results obtained. Given the obvious and reported inter-assay, inter-researcher and inter-laboratory variability in clonogenic survival results of various well-established cell lines, we speculated that these factors might be of major importance for the growth behavior of cells in vitro, for the PE, and, in turn, for the calculated survival results. Here, we provide clonogenic survival data of a large number of non-cooperatively and cooperatively growing cancer cell lines and show that the PE of the latter is far from being constant as it depends on the assay volume per cell during plating. This severely affects the robustness of subsequent survival calculations and generates assay-intrinsic errors which are not amenable to statistical error analyses. Therefore, we propose a novel mathematical approach involving power regression and interpolation of matched colony numbers at different irradiation doses which accounts for the effects of cellular cooperation and allows the extraction of more robust survival results.

## Methods

### Cells and reagents

50 human cancer cell lines of different entities—pancreatic ductal adenocarcinoma, lung adenocarcinoma, glioblastoma, head and neck squamous cell carcinoma, and breast cancer of various subtypes—were purchased from ATCC (Manassas, VA, USA), CLS (Heidelberg, Germany), or the DSMZ (Braunschweig, Germany), and cell line authenticity as well as absence of mycoplasma infection were routinely confirmed (Please see Additional file 1: Table S1 for short tandem repeat profiling of all cell lines). Briefly, all cell lines were cultured in a humidified atmosphere at 37 °C in media containing heat-inactivated fetal calf serum (FCS), 100 U/ml penicillin and 0.1 mg/ml streptomycin. EMEM medium was obtained from CLS, all other culture media and supplements from ThermoScientific (Schwerte, Germany) (For details, please see Additional file 1: Table S2).

### X-ray treatment

Irradiation at the indicated doses was performed using an RS225 X-ray tube (X-Strahl, Camberley, UK) operated at 200 kV and 10 mA (Thoraeus filter, 1 Gy in 242 s).

### Clonogenic survival assays

Single-cell suspensions of exponentially growing cultures were seeded into six-well plates in a range of 7 × 10^0^ to 1 × 10^5^ cells (expecting a resulting range of ≤ 15 to an uncountable number of colonies per well) and allowed to adhere. Upon adherence, cell culture medium was refreshed (2 ml/well in most experiments), and cells were subsequently irradiated at the indicated doses. Depending on the proliferation rate, cells were then incubated at 37 °C for eight to 33 days, and cell growth of all six-well plates of a given cell line was stopped simultaneously.

Fixation and staining were performed using 80% ethanol containing 8‰ methylene blue (Sigma Aldrich, Taufkirchen, Germany). Colonies of ≥ 50 cells were counted under a stereomicroscope. Depending on cell morphology and colony size, counting was performed at 10- to 40-fold magnification.

In some experiments, conditioned cell culture medium from near-confluent culture flasks was used. To this end, 2.5 × 10^6^ BT20 cells or 1 × 10^6^ MDA-MB231 cells were seeded into T175 culture flasks and incubated for 5 or 6 days, respectively. Subsequently, the culture media were centrifuged (314 g, 5 min) and the cell-free supernatants were stored at -80 °C until further use.

### Plating efficiency-based calculation of survival fractions

State-of-the-art, PE-based calculation of survival fractions was performed as recommended [1]. Briefly, PEs were determined by dividing the number of colonies obtained by the number of cells seeded under untreated conditions and were used for normalization of surviving fractions as calculated by dividing the number of colonies obtained by the number of cells seeded at a given radiation dose.

### Calculation of survival fractions by power regression and interpolation of matched colony numbers at different irradiation doses

OriginPro 9.1 (OriginLab Ltd., Northampton, MA, USA) was used for all regression and interpolation analyses. Power regression (C = a × S^b^) was employed to model the number of counted colonies per well (C) in dependence of the number of cells seeded (S) and to determine the coefficient a and the exponent b. Subsequently, interpolation was used to approximate the number of cells that need to be seeded to obtain a given number of colonies (C = 5 to 100). Survival fractions were derived by dividing the number of cells that need to be seeded to obtain C = 5 to 100 colonies under untreated conditions by the number of cells that need to be seeded to obtain identical, matched numbers of colonies upon irradiation at the given doses:

$$C_{0} = a_{0} S_{0}^{{b_{0} }} \;{\text{and}}\;C_{x} = a_{x} S_{x}^{{b_{x} }} ,$$

or

$$S_{0} = \exp \frac{{\log \left( {\frac{{C_{0} }}{{a_{0} }}} \right)}}{{b_{0} }}\;{\text{and}}\;S_{x} = \exp \frac{{\log \left( {\frac{{C_{x} }}{{a_{x} }}} \right)}}{{b_{x} }}.$$

Accordingly,

$$SF = \frac{{S_{0} }}{{S_{x} }} = {\exp}\left( {\frac{{\log \left( {\frac{{C_{0} }}{{a_{0} }}} \right)}}{{b_{0} }} - \frac{{\log \left( {\frac{{C_{x} }}{{a_{x} }}} \right)}}{{b_{x} }}} \right).$$

With

$$C_{0} = C_{x} = C$$

follows

$$SF = \frac{{S_{0} }}{{S_{x} }} = {\exp}\left( {\frac{{\log \left( {\frac{C}{{a_{0} }}} \right)}}{{b_{0} }} - \frac{{\log \left( {\frac{C}{{a_{x} }}} \right)}}{{b_{x} }}} \right),$$

where a_0_ and b_0_ are the estimated parameters of the power regression of the 0 Gy data, a_x_ and b_x_ analogous for x Gy. Accordingly, for every radiation dose up to 96 survival fraction values (for up to 96 different C-values) were obtained and plotted (Fig. 2b).

In order to visualize the impact of cell density on the current state-of-the-art, PE-based survival calculations, this formula was also adapted to non-matched colony numbers within the range of C = 5 to 100 (Fig. 2c):

$$SF = \frac{{\frac{{C_{x} }}{{S_{x} }}}}{{PE_{0} }} = \frac{{\frac{{C_{x} }}{{S_{x} }}}}{{\frac{{C_{0} }}{{S_{0} }}}} = \frac{{C_{x} }}{{C_{0} }}\frac{{S_{0} }}{{S_{x} }} = \frac{{C_{x} }}{{C_{0} }}{\exp}\left( {\frac{{\log \left( {\frac{{C_{0} }}{{a_{0} }}} \right)}}{{b_{0} }} - \frac{{\log \left( {\frac{{C_{x} }}{{a_{x} }}} \right)}}{{b_{x} }}} \right),$$

where PE_0_ is the plating efficiency at 0 Gy.

### Analysis of proliferation rates

Single-cell suspensions were seeded into six-well plates at three different cell densities. Upon adherence, the culture medium (2 ml/well) was refreshed, and cells were incubated for 11 to 17 days reflecting different time points during the clonogenic growth process. Subsequently, cells were washed twice in PBS and detached by trypsinization. The number of cells and the doubling time in each individual well were determined by counting appropriate dilutions in a hemocytometer (Neubauer improved, BRAND, Wertheim, Germany).

### Statistical analyses

Heatmaps of clonogenic survival results were generated using the matrix visualization software Morpheus (https://software.broadinstitute.org/morpheus). OriginPro 9.1 was used for all statistical procedures. Group comparisons were performed using paired exact Wilcoxon signed rank tests, or one-way Kruskal–Wallis-ANOVAs as indicated.

## Results

**Fig. 1:**
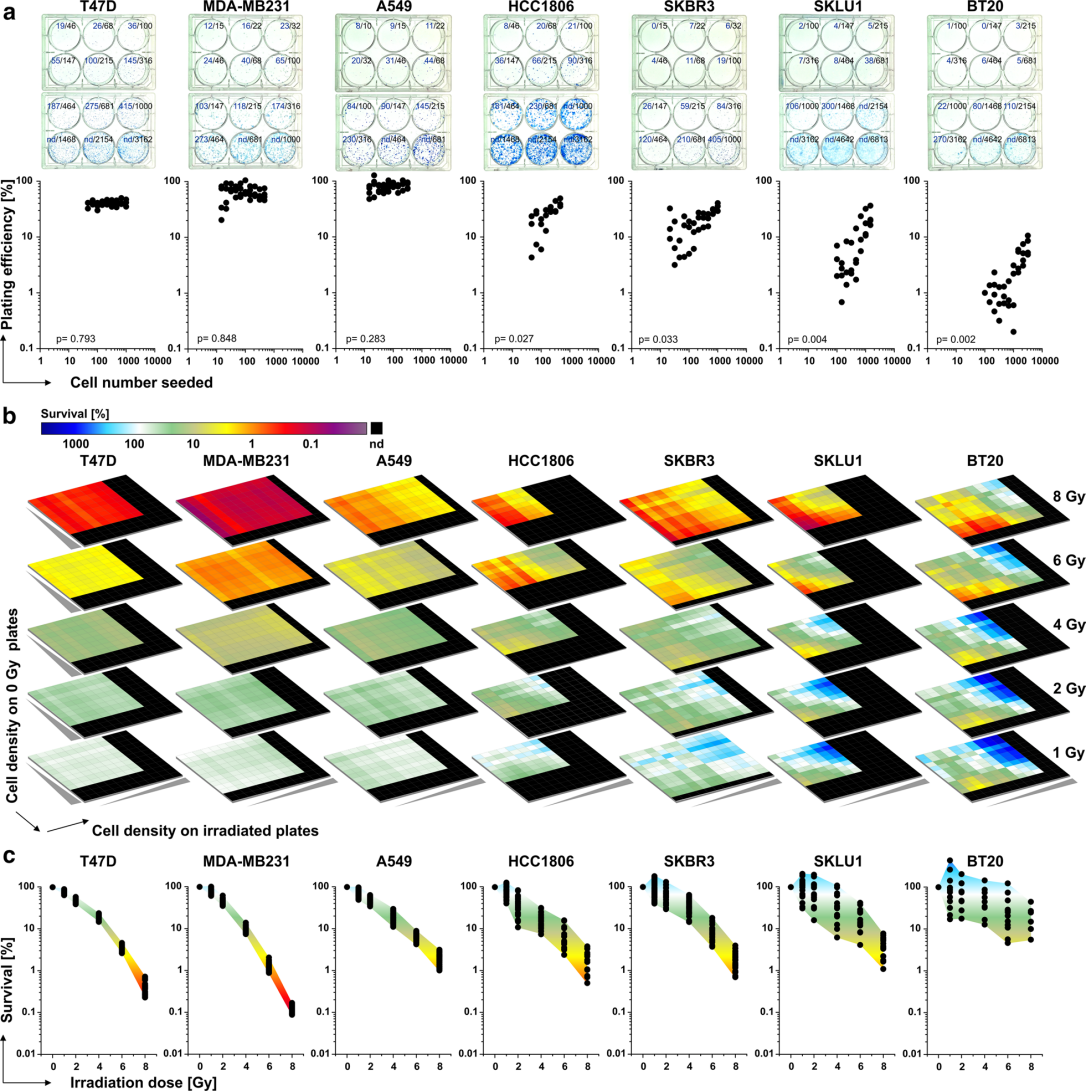
Clonogenic survival of seven different cancer cell lines as determined by the conventional PE-based algorithm. For each cell line, geometric series of twelve different cell densities were seeded for each radiation dose anticipating a resulting range of ≤ 15 to an uncountable number of colonies per well (0–8 Gy; three (T47D and HCC1806) to four independent biological replicates (all other cell lines)). **a** Representative images of untreated plates with the number of single cells seeded in black and the number of colonies obtained in blue (upper part). The PEs derived from all countable wells are given below. *p*-values were calculated by one-way Kruskal–Wallis ANOVA with the factor "cell number seeded". **b** Heatmap of clonogenic survival results as calculated from the mean values of counted colonies at all countable cell densities according to the PE-based method. Note that the number of cells seeded varies between cell line and radiation dose. **c** Reduced range of results from b if only wells with 5 to 100 counted colonies are considered for calculation.

We initially performed clonogenic survival assays with a panel of seven cell lines and calculated the PEs obtained when plating single-cell suspensions at different cell densities without further treatment (Fig. 1a). The results for MDA-MB231, T47D, and A549 cells were virtually constant over a wide range of cell numbers seeded. However, the PEs of the four other cell lines increased by one to two orders of magnitude with increasing numbers of cells seeded, thus challenging the concept of cell line-specific, constant PEs.

Next, we assessed clonogenic survival upon irradiation using the PE-based normalization algorithm which represents the current state-of-the-art. For each radiation dose, an array of clonogenic survival results was calculated by normalizing the surviving fractions from each countable cell density on the irradiated plates to the spectrum of PEs obtained from each countable cell density on the control plates (Fig. 1b). As expected, the results for MDA-MB231 and T47D cells were rather stable irrespective of the cell numbers seeded. On the contrary, the range of clonogenic survival results at a given radiation dose was considerably larger for the other cell lines and varied over several orders of magnitude in case of BT20 and SKLU1 cells. We now assumed that experienced researchers would only consider wells with intermediate numbers of colonies (C = 5 to 100) for analysis. Although this clearly reduced the variance, the range of calculated results that could be extracted from the given dataset still remained unsatisfyingly wide for several cell lines (Fig. 1c).

**Fig. 2:**
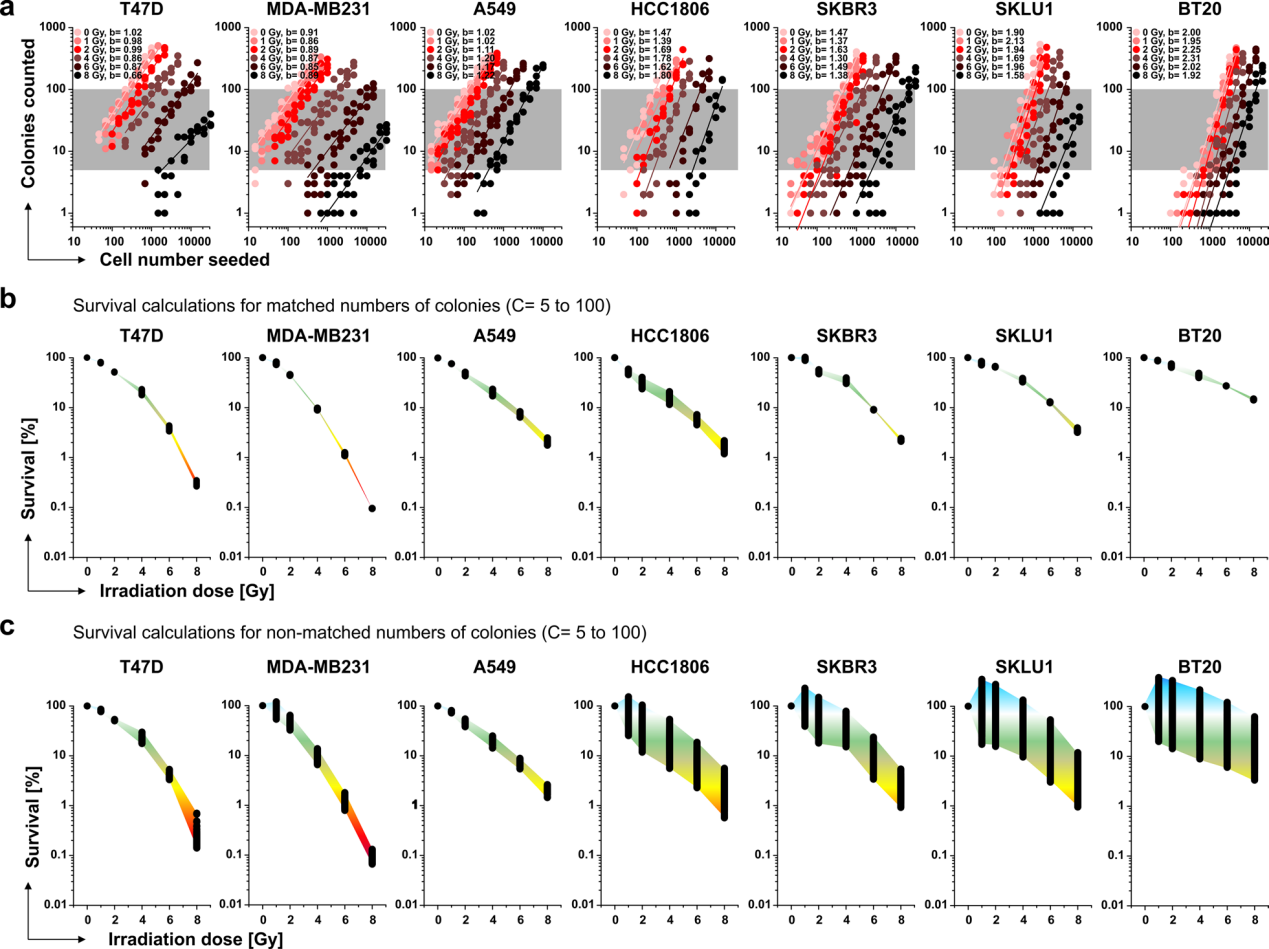
Clonogenic survival of seven different cancer cell lines as determined by the novel approach involving power regression and interpolation. The dataset shown in Fig. 1 was used for analyses. **a** Double-logarithmic presentation of the clonogenic survival data. Power regression lines (C = a × S^b^) for the number of colonies counted (C) in dependence of the number of cells seeded (S) are superimposed. The values for the exponents b are indicated for each fit. **b** Range of survival results as determined by the novel approach simulating wells with matched numbers of colonies C = 5 to 100 at different irradiation doses (grey areas depicted in **a**) and displayed according to Fig. 1c. **c**, Range of survival results as determined by the novel approach simulating wells with non-matched numbers of colonies C = 5 to 100 (grey areas depicted in **a**) as implied in the PE-based method and displayed according to Fig. 1c

To overcome this inadequacy, we reanalyzed the dataset using a novel mathematical approach that accounts for the effects of cellular cooperation by adapting the cell numbers seeded to the cell line-specific power parameter of the colony formation function: First, we performed power regression analysis for the number of colonies obtained (C) versus the number of cells seeded (S) (C = a × S^b^; note that on a double-logarithmic scale as shown in Fig. 2a, any power function appears approximately linear with b determining the steepness of the regression line). As anticipated by the results from Fig. 1a, the relationship between C and S reached from nearly linear (b ≈ 1, e.g. for T47D cells) to more than quadratic (b > 2, e.g. for BT20 cells) reflecting a relevant impact of cellular cooperation on clonogenic growth for cells with b-values > 1. Second, we employed the power regression results to interpolate the number of cells to be seeded to yield identical, matched numbers of colonies and determined the clonogenic survival fractions by calculating the S_0_/S_x_ ratios. In analogy to Fig. 1c, we performed this analysis for C = 5 to 100 and observed a clear reduction in variance of the survival results derived from the same dataset as compared to the conventional PE-based method (Fig. 2b). In order to visualize the impact of cell density more stringently, we also performed the survival calculations according to our workflow with non-matched numbers of colonies for C = 5 to 100 as implied in the current PE-based model (Fig. 2c). Expectedly, the obtained survival results revealed similar ranges of variation as in case of the empirical, PE-based calculations (Fig. 1c). For cells with cooperative growth behavior this impact of cell density on the range of calculated results was particularly evident. In strong contrast, survival calculations based on matched numbers of colonies at different irradiation doses clearly compensated the effect of cell density and allowed the extraction of more robust results. Hence, in terms of robustness regarding the impact of cell density and cellular cooperation, power regression and interpolation of matched colony numbers at different irradiation doses were clearly superior to the current state-of-the-art, PE-based algorithm [1].

**Fig. 3:**
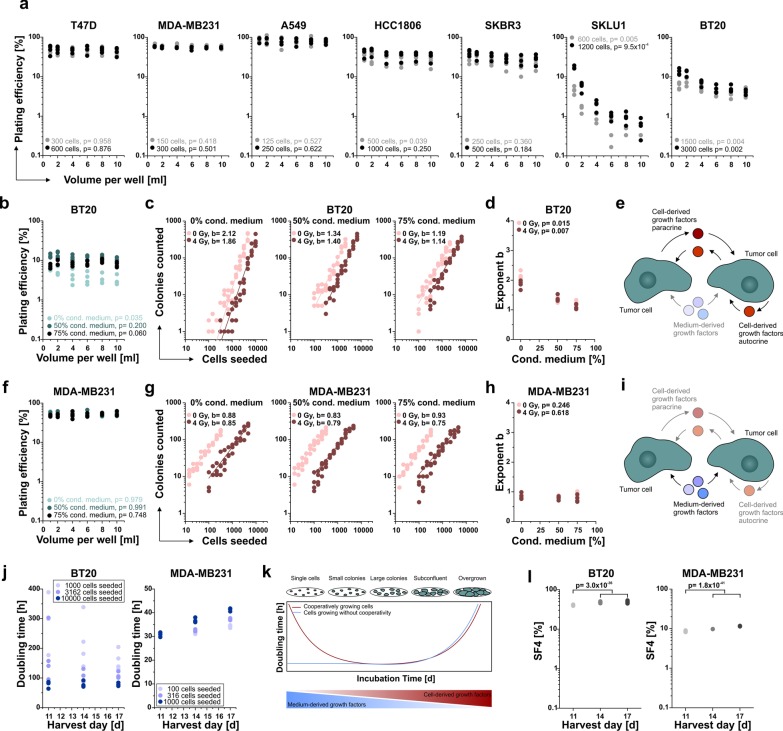
Cellular cooperation is driven by soluble growth supporting factors. **a** Analysis of PE in dependence of the assay volume for all cell lines used in Fig. 1. Four independent biological replicates are presented for all cell lines except MDA-MB231 (three replicates), and *p*-values were calculated by one-way Kruskal–Wallis ANOVA with the factor “volume per well”. **b–d** BT20 cells were cultured in standard medium supplemented with 0, 50 or 75% of conditioned medium collected from subconfluent BT20 cultures (four independent biological replicates). **b** PE of BT20 cells grown in different assay volumes. *p*-values were determined by one-way Kruskal–Wallis ANOVA with the factor “volume per well”. **c** Double-logarithmic presentation of clonogenic survival data from BT20 cells. Power regression lines (C = a × S^b^) are superimposed, and values of the exponents b are displayed. **d** Visualization of the b-values as calculated in **c**. *p*-values were determined by one-way Kruskal–Wallis ANOVA with the factor “conditioned medium [%]”. **e** Scenario of cooperative growth behavior depending on the production of soluble growth promoting factors. **f–h** MDA-MB231 cells were used for the same experiments as shown for BT20 in **b**–**d** (four independent biological replicates). **f** PE of MDA-MB231 cells in different assay volumes. *p*-values were determined as in **b**. **g** Double-logarithmic presentation of clonogenic survival data from MDA-MB231 cells with power regression lines and b-values as in **c**. **h** Visualization of the b-values as calculated in **g**. *p*-values were determined as in **d**. **i** Scheme of non-cooperative cell growth independent of cell-derived factors. **j** Analysis of population doubling times of BT20 and MDA-MB231 cells harvested after different incubation periods. Four independent biological replicates plated in averaged technical duplicates are shown. **k** Schematic overview on the growth behavior of cooperatively and non-cooperatively growing cells in vitro. **l** SF4 values for BT20 and MDA-MB231 for different incubation periods. Calculations were performed by power regression and interpolation as in Fig. 2 and *p*-values were obtained by one-way Kruskal–Wallis ANOVA with the factor “11d vs. longer incubation times”

Given the unexpectedly high b-values of four out of seven cell lines, we aimed to dissect the underlying mechanisms of this non-linear increase in colony number with the number of cells seeded. Puck et al. had already described the "loss by diffusion of any necessary metabolites escaping from the cells" as a factor which impairs the PE [12]. In order to assess the contribution of diffusion phenomena to clonogenic growth, we plated identical numbers of cells and incubated them in different volumes of culture medium. Intriguingly, a significant volume-dependent decline in PE was seen for the cell lines with the highest b-values (Fig. 3a). Moreover, when the culture medium was supplemented with conditioned cell-free medium of near-confluent cell cultures from cooperatively growing BT20 cells, the volume-dependent decrease in PE was completely eliminated (50% conditioned medium) or even trended to be inverted (75% conditioned medium) (Fig. 3b). In line with these findings, the b-values of both untreated and irradiated BT20 cells declined significantly when using conditioned media (Fig. 3c, d). These data illustrate that the clonogenic capacity of cooperatively growing cells is largely dependent on the concentration of soluble cell-derived factors, whereas growth factors initially present in the culture medium per se are not sufficient to adequately sustain cell growth in this scenario (Fig. 3e). In contrast, conditioned media from non-cooperatively growing MDA-MB231 cells did not significantly affect the PE of single cells of the same type, and we did not observe a relevant impact on the approximately linear correlation between the number of cells seeded and the number of colonies obtained (Fig. 3f–h). Hence, in the case of non-cooperatively growing cells, the growth factors in the culture medium are sufficient to enable cell growth, and auto-/paracrine growth stimulation does not further enhance the clonogenic capacity (Fig. 3i).

Considering the very different growth behavior of single cells of different cell lines (Fig. 3e, i), we hypothesized that the extent of auto-/paracrine growth stimulation may also influence the proliferation rate of the cells and therefore investigated to which extent this affects the results of clonogenic survival assays. To this end, single-cell suspensions of BT20 and MDA-MB231 were plated, incubated for 11 to 17 days, and the total amount of adherent cells at three different seeding densities was determined. In accordance with its high b-value, the population doubling time of BT20 cells was dependent on the number of cells seeded into the culture dishes (Fig. 3j, left panel): The population doubling time of the low-density wells (1000 cells initially seeded) constantly declined with increasing assay incubation time but remained higher than in culture dishes seeded with a tenfold higher number of cells over time. These findings show that there are large differences in doubling times of cooperatively growing cells within the very same assay which skew the clonogenic survival fractions calculated. In contrast, non-cooperatively growing MDA-MB231 cells initially displayed proliferation doubling times of approximately 30 h on day 11—irrespective of the cell density seeded. However, the higher-density culture dishes finally reached a subconfluent stage (d17) where cell proliferation was most likely impeded by increasing contact inhibition and lack of nutrients (Fig. 3j, right panel). These data confirm that the influence of cell seeding density on the proliferation rate of non-cooperatively growing cells is virtually neglectable—at least within a reasonable range of cell numbers seeded which do not reach subconfluency during incubation. Very similar observations as those from Fig. 3j were obtained with the two lung adenocarcinoma cell lines used in Figs. 1 and 2. In full accordance with their b-value of 1.02, non-irradiated A549 cells recapitulated the findings for MDA-MB231 cells, whereas the growth behavior of untreated SKLU1 cells (b = 1.90) was comparable to cooperatively growing BT20 cells (data not shown).

Taken together, our experiments show that cell proliferation in single cell settings in vitro is a highly dynamic process with clear differences between cell lines raising concerns about the robustness of PE-based approaches which obviously compare culture dishes at very different stages of clonogenic growth (Fig. 3k).

Since the stopping time point of clonogenic assays is also dependent on the researchers’ experiences with different cell lines and personal preferences regarding colony counting conditions, we also tested whether the overall incubation period impacts the survival fraction results obtained. In spite of the small degree of variation of the SF4 values derived by the novel approach, we found an overall small but significant effect of the total incubation time on the SF4s obtained (Fig. 3l). For both cell lines, an 11-day incubation resulted in lower SF4 values compared to intermediate (d14) and late (d17) incubation periods. Apparently, for many potentially clonogenic cells on the irradiated plates, 11 days were an insufficient time period to repair radiation damage and restart proliferation to finally reach a colony size of > 50 cells. This observation underlines the necessity to evaluate both irradiated and control plates in order to determine a suitable stopping time point of an experiment.

**Fig. 4:**
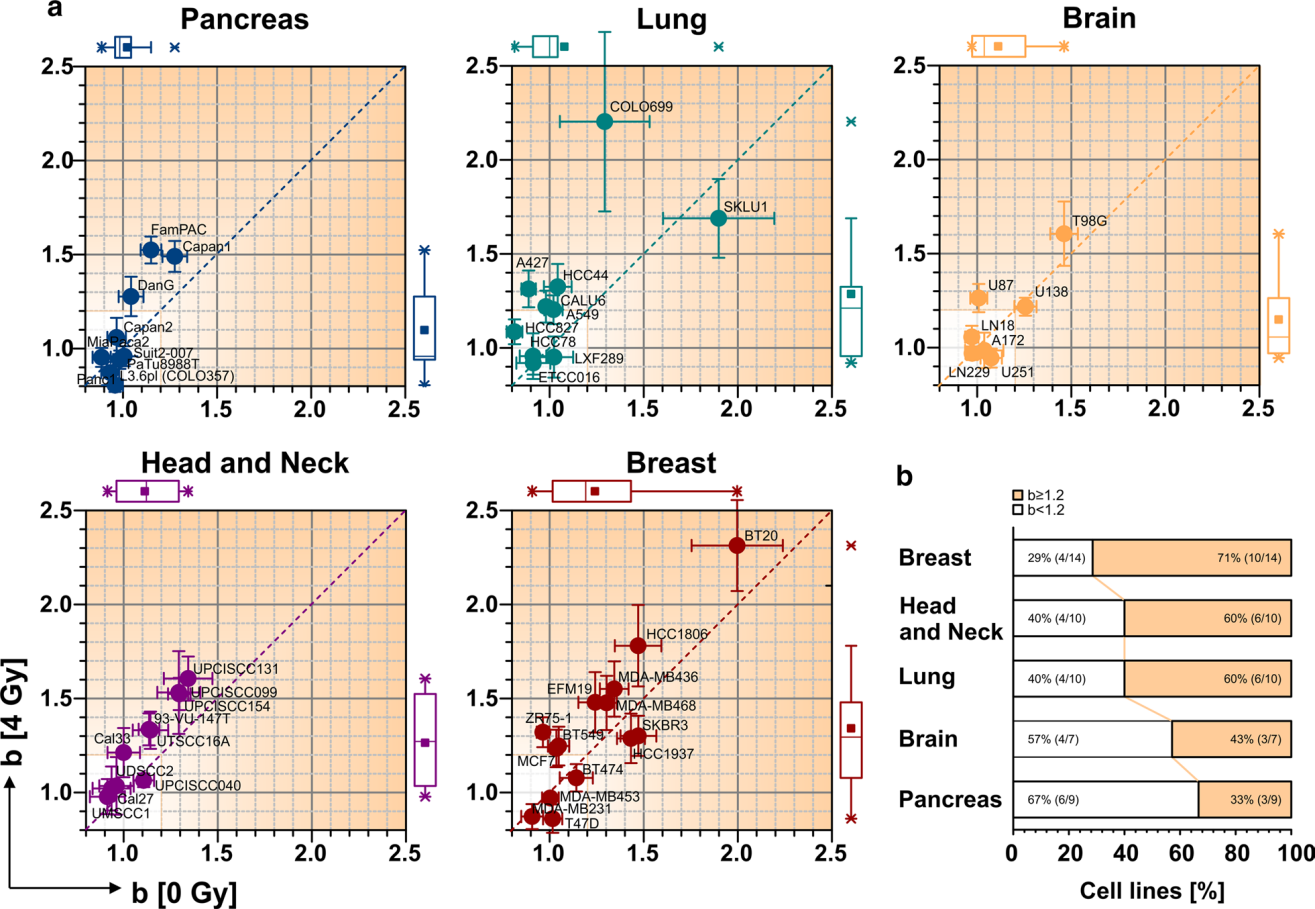
Cellular cooperation is a very common phenomenon observed in various cell lines across different cancer entities in vitro. **a** Geometric series of twelve different cell densities were seeded for various cancer cell lines of the pancreas (blue), lung (turquoise), brain (orange), head and neck (violet), and breast (dark red) and combined with the data from Fig. 1 (Three to five independent biological replicates per cell line). For each cell line, a pair of b-values (0 Gy vs. 4 Gy) is shown in the graph of the respective tumor entity. Areas highlighted in pale brown indicate cooperative growth behavior resulting in less than borderline robustness if analyzed by PE-based approaches. Error bars represent standard errors of fitted b-values, and boxplots for entity-specific distributions of b-values are shown at the edges of the graphs. **b** Bar diagrams indicating the percentage of cell lines displaying a relevant degree of cellular cooperation in vitro. Data taken from **a**

In view of the very broad use of clonogenic assays, we finally assessed the prevalence of cellular cooperation in a panel of 50 commercially available cell lines of five different tumor entities (pancreatic ductal adenocarcinoma, lung adenocarcinoma, glioblastoma, head and neck squamous cell carcinoma, and breast cancer of various subtypes) under untreated conditions as well as after irradiation at 4 Gy (Fig. 4). Strikingly, cooperative growth behavior was found in various cell lines across all tumor entities analyzed. The phenomenon was most pronounced within the set of breast cancer cell lines, whereas pancreatic cancer cell lines were, overall, less affected. Nevertheless, individual, strongly cooperative cell lines with b-values > 1.5 were found in all tumor entities, thus underlining the relevance of this phenomenon for clonogenic survival analyses irrespective of the cancer subtype analyzed.

Referring back to the data presented in Fig. 1, it becomes obvious that PE-based survival analyses for the lung cancer cell line A549 with weakly cooperative growth preferentially upon irradiation (b = 1.02 − 1.22, see Fig. 2a) showed borderline dependence on cell density. Hence, it can be concluded that PE-based analyses will result in rather unstable results for any experiment where cellular cooperation gives rise to b-values of ~1.2 or higher at any of the radiation doses analyzed. In the multi-entity cell line panel, this applied to 28 of 50 cell lines (Fig. 4b). Taken together, these findings indicate that cellular cooperation is an unexpectedly common phenomenon across various cell lines of different cancer entities which strongly impedes the robustness of clonogenic survival calculations when not properly taken into account. In contrast to PE-based survival analyses, the approach involving power regression and interpolation of matched colony numbers at different irradiation doses mathematically counterbalances the effects of cellular cooperation and allows the extraction of more robust survival results.

## Discussion

The clonogenic assay has been used in numerous studies to quantify clonogenic growth and its abrogation by cytotoxic stimuli, including radiation, chemotherapeutic drugs, and/or molecularly targeted agents, in vitro. The current standard procedure to determine survival fractions is based on the assumption that clonogenic growth in treated cell cultures can be normalized to the untreated controls via dividing by a cell line-specific, constant PE.

Here we show, however, that this is not universally applicable. In contrast, our data clearly indicate that the correlation between the number of cells seeded in a culture dish and the number of colonies obtained is far from always being linear. For cell lines with cooperative behavior, the PE-based analysis of clonogenic survival data yielded results with large to enormous assay-intrinsic errors. Even if only culture dishes with reasonable numbers of colonies (C = 5 to 100) were used for analysis, clonogenic survival fractions at a given dose differed by far more than one order of magnitude for cell lines with high degrees of cellular cooperation. Of note, virtually any survival curve (steep or flat, moderately or strongly curved, linear, quadratic, or irregular) can be derived from this range of results calculated from the given dataset—an observation which might be of particular importance for radiation biologists.

Taken together, our data show that conventional PE-based analysis of clonogenic survival data performs inappropriately as soon as cellular cooperation occurs under one or more conditions within an experiment, and extracted survival results will vary within an unsatisfyingly large range. Specifically, the results will be heavily skewed if only one or few similar cell densities are plated. This practice generates assay-intrinsic errors which are a direct consequence of the chosen cell densities and therefore not amenable to statistical error analyses. For cooperatively growing cell lines, our observations may partly explain reported inter-assay, inter-researcher, and inter-laboratory incongruences of treatment response data [13]. A meta-analysis of A549 colony formation assay data further supports this hypothesis: Within a panel of 156 different studies, Nuryadi et al. reported on SF4 values for this specific cell line ranging from 5 to 90% with an SF4 interquartile range of more than 25% [14]. Although diverse other parameters may certainly influence treatment response data, we conclude from our data that cellular cooperation is a major factor explaining inter-study variability. Since even small differences in clonogenic survival fractions may encourage researchers to postulate and study new scientific hypotheses that might eventually be based on false precision, we developed a novel analysis approach which is less susceptible to the impact of cell density—especially but not only for cooperatively growing cell lines. This method accounts for non-linear relationships between cell numbers seeded and colony numbers obtained by scoring culture dishes with a wide range of cell numbers seeded for all treatment conditions.

Mathematically, our approach utilizes power regression and interpolation of matched numbers of colonies at different irradiation doses. Applied to the very same dataset that was used for PE-based calculations it provided clearly more stable, cell density-independent results. Attentive readers may have noticed that the survival fraction calculations performed according to the method presented here, rely solely on the coefficient a and the exponent b as extracted by power regression. Although this obviously compensates for the effects of cellular cooperation, it bears another quality of error which derives from the inaccuracy of regression and which cannot be quantitatively compared to the similar quality of error in PE-based survival fraction calculations. Accordingly, this error should be minimized by ensuring careful experimental design with a sufficient number of independent replicates. Moreover, survival fraction calculations should only be performed with power regression results of proper performance as indicated by the regression coefficient R.

Our mathematical approach basically replaces PE-based clonogenic survival calculations by the question:

### How many times more cells need to be seeded into a treated culture dish to yield the identical number of colonies as in a control dish?

The exponent b is of particular importance in this regard. It indicates whether the correlation between the number of seeded cells and the number of counted colonies is linear (b ≈ 1) or not. High b-values, as obtained for BT20 and SKLU1 cells, indicate that cell growth in vitro is decelerated (or entirely abrogated) if the volume of culture medium per cell is increased—either by use of large assay volumes or reduction of the number of cells seeded. It should be emphasized that b-values are by no means specific for a certain cell line but rather a consequence of the chosen cell culture medium, several assay incubation parameters, and the experimental procedure including virtually any aspect that might affect the clonal outgrowth of cells which are in an extreme stress situation when plated as single cells, such as medium formulation, supplementation with nutrients and growth factors, methods used for cell separation, plasticware, etc.. For instance, use of conditioned media from near-confluent BT20 cells strongly attenuated the cooperative behavior of BT20 single cells, whereas this procedure had no impact on the clonogenic growth of non-cooperatively growing MDA-MB231 cells. Furthermore, the doubling time of cooperative BT20 cells was dependent on both assay incubation time and cell density in the well, thus giving a self-evident biological explanation for imprecise clonogenic survival fractions obtained by PE-based calculations: A proliferating cell cluster’s growth rate may simply be too slow to reach the threshold of 50 cells per colony within the assay incubation time. Hence, the apparent "non-clonality" of a cluster of e.g. 35 slowly proliferating cells at the stopping time point is merely an inevitable consequence of the assay incubation time which is—at least to a certain extent—chosen arbitrarily. In this context, we additionally analyzed the impact of the incubation time on clonogenic survival fractions obtained and observed that it is insufficient to determine the stopping time point by inspection of the control dishes alone as suggested by others [1]: Premature termination of the incubation period may lead to exceedingly low survival fractions on plates with more aggressive treatment where damage repair before continuation of cell growth requires additional time.

Importantly, our data are fully in line with seminal findings of pioneering cell culture researchers in the 1940s and 1950s and simply reflect a phenomenon which was under extensive investigation at that time. Puck and colleagues were the first to publish a survival curve of irradiated single cells in 1956. However, the biggest scientific challenge to this fundamental achievement was an at that time unresolved problem of mammalian cell culture: Cell lines stopped growing in vitro as soon as the cells were plated at low density. An attempt to overcome this problem was made in 1948 by Sanford et al., who succeeded at growing single-cell-derived fibroblast colonies in small capillaries where diffusion of cell-derived factors into the medium was strongly reduced, thus allowing sufficient autocrine growth stimulation [15]. They identified the importance of pre-conditioning the culture medium by cultivated cells and concluded that a cell culture medium sufficient to allow infinite growth of high-density cell culture is in fact “far from optimal for the growth of a single cell”. In line with this, Earle et al. described that plating the respective cell type at very low density resulted in cell death [16], and this work formed the basis for the first publication on clonogenic growth of mammalian cells in vitro by Puck and Marcus in 1955 [17]. Inspired by the need of conditioned culture medium to facilitate single-cell growth, they used a co-culture system of HeLa single cells and a layer of heavily irradiated feeder cells of the same type. In agreement with the preceding studies, they concluded that the inhibition of single-cell growth in large assay volumes was due to the “loss of a short-lived, diffusible factor” [17]. In later publications, such as the one with the first survival curve of irradiated mammalian cells, Puck and colleagues frequently omitted the use of feeder layers, since they had developed advanced culture techniques allowing single-cell growth with 100% PE without growth factor supplementation by feeder cells [2, 12]. They stated that careful washing and trypsinization protocols were essential in this regard [12] and coined the term “cooperative action” to describe that cells in a culture dish may differ with regard to genotype as well as physiological state [18]. Our findings recapitulate these observations: Within a 50 cancer cell line panel, we observed that suboptimal growth of single cells in modern, standardized culture media supplemented with FCS is still a very common phenomenon as can be deduced from the finding that more than half of the cell lines displayed cooperative growth behavior. Hence, if suboptimal PEs are found for a certain cell line, the clonogenic assay is likely to simultaneously detect both the influence of the treatment of interest *and* the impact of cellular cooperation. It was not in the scope of this study to identify specific growth supporting factors which might affect the PE of the cell lines analyzed. However, we hypothesize that suboptimal growth conditions for single cells of a given cell line may result from very different parameters, such as low concentrations of classical growth factors and/or hormones (e.g. epidermal growth factor or estrogen) but also various low- and high-molecular weight metabolites for which at least a fraction of single cells displays auxotrophy. Moreover, nutrient supplementation of single cells in a culture dish will likely be influenced by physicochemical parameters of the surrounding medium and the plasticware, including the degree of protein binding of the respective auxotrophic factors or their adsorption to the plastic surface. In theory, this problem could be addressed by taking measures that restore the maximum PE in low-density conditions so that a linear correlation between S and C is (re-)established (b = 1). Puck’s recommendations for the use of feeder cells, conditioned media, and/or embedding single cells into soft agar may be sufficient to achieve this for selected cell lines and should increase the robustness of PE-based calculations accordingly. However, it is obvious that it can be more than challenging to refine and standardize the assay conditions so that single cell survival and growth rates are optimal for every single cell type of interest [19]. We decided to accept suboptimal assay conditions for single cell growth and instead developed a computational method for clonogenic survival data analysis which accounts for this well-described phenomenon. Obviously, our approach using power regression and interpolation was beyond the technical capabilities of the 1950s when survival data were fitted by eye [20]. However, somehow the relevance of cellular cooperation moved out of focus during the following decades. Although a few reports on non-linearity in colony formation assays were reported over time, the limited performance of PE-based analyses was not addressed [21–24].

Interestingly, these studies reported on a less-than-linear increase in colony numbers with increasing numbers of seeded cells for certain cell types under specific conditions. In accordance with this, for a few cell lines in our panel we also obtained b-values slightly below 1.0. Three different scenarios may explain this observation, of which two are due to methodological artifacts: Firstly, b-values slightly below 1.0 may result from counting wells with a large number of overgrown colonies where small colonies are overlooked by the researcher (see wells marked with “nd” in Fig. 1a). Secondly, cell growth of dishes with high cell numbers may be inhibited in rather early stages due to a rapid decline in nutrient concentration thus resulting in abortive colonies. A third—and biologically less intuitive—option is competitive behavior of cell growth, for instance due to secretion of growth inhibitory factors. Importantly, any of these phenomena is accounted for by the regression and interpolation approach, because it considers any deviation from linearity as reflected by the b-value.

Moreover, it is remarkable that the b-values of various cell lines for untreated compared to irradiated conditions are not identical. In the majority of these cases, b-values of irradiated cells tend to be higher than the respective b-values of untreated controls, indicating that cellular cooperation increases upon irradiation. Consequently, the range of survival fraction values obtained for C = 5 to 100 colonies becomes wider than in case of nearly identical b-values (see cell lines HCC1806 and A549). This implies that it is technically not possible to extract more precise survival values by means of the clonogenic assay procedure—unless one fixed number of colonies (C) was selected for analysis. Furthermore, cell lines with exceedingly high b-values for treated cells may be of particular interest with regard to therapy resistance studies. For instance, radiation-induced survival factor(s) secreted by a certain cell type might be identified due to a correspondingly high b-value.

In summary, our data show the need to carefully analyze data from colony formation experiments and to consider the underestimated impact of cellular cooperation on survival fraction calculations. This may greatly increase the reliability of the clonogenic assay—and the resilience of any hypothesis based on it.

## Conclusions

Here, we demonstrate that cellular cooperation considerably skews plating efficiency-based calculations of clonogenic survival results of various established cancer cell lines. PE-based survival calculations exhibit a high degree of false precision unless linearity between the numbers of cells seeded and colonies obtained is proven for control as well as treated culture conditions. If cellular cooperation is not properly accounted for, assay-intrinsic errors can exceed one order of magnitude and are not amenable to statistical error analysis. Accordingly, PE-based survival results of cell lines displaying high degrees of cellular cooperation can at best be considered semiquantitative.

In order to address this challenge, we propose a novel mathematical approach involving power regression and interpolation of matched colony numbers at different irradiation doses which conceptionally removes the disruptive effect of cell density and cellular cooperation almost completely. For future studies, we see the need and strongly recommend to replace PE-based calculations by the method presented here.

## Supplementary information

**Additional file 1: Table S1.** Short tandem repeat profiling of cell lines used in this study (Service provided by DSMZ, Braunschweig). **Table S2.** Overview on cell lines and culture media used in this study. FCS (fetal calf serum), P/S (100 U/ml penicillin and 0.1 mg/ml streptomycin).

## Data Availability

The datasets generated and analyzed during the present study are available from the corresponding author on reasonable request.

## Abbreviations

FCS: Fetal calf serum
PE: Plating efficiency
P/S: Penicillin/streptomycin
SF: Surviving fraction
