# Supplementary material for: The clonogenic assay: robustness of plating efficiency-based analysis is strongly compromised by cellular cooperation
Source: Radiat Oncol. 2020 Oct 29;15:248. doi: 10.1186/s13014-020-01697-y (PMC7597001; doi:10.1186/s13014-020-01697-y)
Supplement: Supplementary file 1 — Additional file 1: Table S1. Short tandem repeat profiling of cell lines used in this study (Service provided by DSMZ, Braunschweig). Table S2. Overview on cell lines and culture media used in this study. FCS (fetal calf serum), P/S (100 U/ml penicillin and 0.1 mg/ml streptomycin). [file 13014_2020_1697_MOESM1_ESM.pdf]

Supplemental Tables

Cell line	D5 D5'		D13 D13'		D7 D7'		D16 D16'		vWA vWA'		TH01 TH01'		TPOX TPOX'		CSF1 CSF1'		Amel Amel'	
Capan1	11	11	9	9	10	11	13	14	16	16	6	6	8	11	11	11	X	X
Capan2	11	12	11	12	9	11	9	13	17	17	9,3	9,3	8	8	11	12	X	X
DanG	12	13	8	8	10	13	8	11	16	18	9,3	9,3	10	10	13	13	X	X
FamPAC	10	11	8	8	11	11	14	14	15	15	9	9	8	8	10	10	X	X
L3.6pl (COLO357)	11	11	8	8	8	10	9	12	18	18	7	7	8	8	11	12	X	X
MiaPaca2	12	13	12	13	12	13	10	13	15	15	9	10	9	9	10	10	X	X
Panc1	11	13	11	11	8	10	11	11	15	15	7	8	8	11	10	12	X	X
PaTu8988T	11	13	12	13	7	8	11	12	16	16	6	6	8	11	11	13	X	X
Suit2 (subclone 007)	11	12	9	11	9	11	9	9	17	17	6	6	8	10	11	12	X	X
A427	12	12	11	12	8	12	11	13	17	17	9	9	8	11	10	12	X	Y
A549	11	11	11	11	8	11	11	12	14	14	8	9	8	11	10	12	X	Y
CALU6	11	11	11	11	10	10	13	13	17	17	9	9	8	8	12	12	X	X
COLO699	11	12	11	11	11	13	12	12	17	17	9	9	8	8	10	10	X	X
ETCC016	11	12	10	10	9	12	12	12	17	19	9	9	8	11	10	12	X	X
HCC44	11	13	11	11	8	10	9	13	15	15	9	9	8	12	9	11	X	X
HCC78	11	12	8	12	9	9	11	13	15	18	7	9	8	11	11	12	X	Y
HCC827	12	12	9	9	11	12	12	12	18	18	6	6	8	8	11	11	X	X
LXF289	9	10	9	11	10	10	13	13	17	18	6	9	11	11	12	13	X	Y
SKLU1	11	11	10	10	9	9	8	8	16	17	7	7	8	10	10	10	X	X
A172	11	12	11	11	11	11	12	12	20	20	6	9,3	8	11	9	12	X	Y
LN18	11	13	12	13	8	10	11	13	17	18	9	9	8	8	12	12	X	Y
LN229	11	12	10	11	8	11	12	12	16	19	9,3	9,3	8	8	12	12	X	X
T98G	10	12	13	13	9	10	13	13	17	20	7	9,3	8	8	10	12	X	Y
U87	11	12	8	11	8	9	12	12	15	17	9,3	9,3	8	8	10	11	X	X
U138	11	11	9	11	9	9	12	13	18	18	6	6	8	8	12	12	X	Y
U251	11	12	10	11	10	12	12	12	16	18	9,3	9,3	8	8	11	12	X	Y
Cal27	11	12	10	11	10	10	11	12	14	17	6	9,3	8	8	10	12	X	X
Cal33	11	12	8	13	8	10	11	11	17	17	9	9	8	8	11	12	X	Y
UDSCC2	10	11	8	8	8	9	11	13	15	18	8	9	8	10	11	12	X	Y
UMSCC1	10	13	8	11	9	12	12	13	15	18	6	6	8	11	10	12	X	X
UPCISCC040	10	11	8	12	9	10	11	11	17	18	6	8	11	11	12	12	X	Y
UPCISCC099	11	12	8	13	7	9 / 10	11	12	14	18 / 19	6	9	11	11	11	12	X	X
UPCISCC131	11	11	11	13	11	12	13	13	15	17	7	9	11	11	12	12	X	Y
UPCISCC154	11	12	9	12	9	10	13	13	17	17	7	7	8	9	10	12	X	Y
UTSCC16A	12	14	9	12	9	11	11	11	19	19	9	9	9	9	10	11	X	X
93-VU-147T	11	12	12	12	10	11	9	11	18	18	7	9	9	11	11	12	X	X
BT20	12	12	11	11	10	10	11	14	16	17	7	9,3	11	11	12	12	X	X
BT474	11	13	11	11	9	12	9	11	15	16	7	7	8	8	10	11	X	X
BT549	11	11	11	11	9	10	8	8	15	15	9,3	9,3	8	8	10	12	X	X
EFM19	11	11	8	12	9	9	11	12	14	14	7	8	8	12	9	9	X	X
HCC1806	13	13	11	11	10	12	10	10	16	18	8	8	8	9	12	12	X	X
HCC1937	12	12	13	13	9	10	13	14	16	17	6	6	11	11	12	12	X	X

MCF7	12	12	11	11	8	9	11	12	14	15	6	6	9	12	10	10	X	X
MDA-MB231	12	12	13	13	8	9	12	12	15	18	7	9,3	8	9	12	13	X	X
MDA-MB436	13	13	10	10	10	10	9	9	14	20	9,3	9,3	8	8	12	12	X	X
MDA-MB453	11	11	12	12	10	10	9	9	17	18	6	6	10	10	10	12	X	X
MDA-MB468	12	12	12	12	8	8	9	9	18	18	7	7	8	9	12	12	X	X
SKBR3	9	12	11	12	9	12	9	9	17	17	8	9	8	11	12	12	X	X
T47D	12	12	12	12	11	11	10	10	14	14	6	6	11	11	11	13	X	X
ZR75-1	13	13	9	9	10	11	11	11	16	18	7	9,3	8	8	10	11	X	X

Supplementary Table S1: Short tandem repeat profiling of cell lines used in this study (Service provided by DSMZ, Braunschweig).

Cell line	Tumor type	Culture medium and supplements	CO_2 [vol%]
Capan1	Pancreatic cancer	RPMI-1640 + 10% FCS + 10 mM HEPES + P/S	5
Capan2	Pancreatic cancer	DMEM + 10% FCS + P/S	7.5
DanG	Pancreatic cancer	RPMI-1640 + 10% FCS + 10 mM HEPES + P/S	5
FamPAC	Pancreatic cancer	RPMI-1640 + 10% FCS + 10 mM HEPES + P/S	5
L3.6pl (COLO357)	Pancreatic cancer	DMEM + 10% FCS + P/S	7.5
MiaPaca2	Pancreatic cancer	DMEM + 10% FCS + P/S	7.5
Panc1	Pancreatic cancer	DMEM + 10% FCS + P/S	7.5
PaTu8988T	Pancreatic cancer	DMEM + 10% FCS + P/S	7.5
Suit2 (subclone 007)	Pancreatic cancer	DMEM + 10% FCS + P/S	7.5
A427	Lung adenocarcinoma	RPMI-1640 + 10% FCS + 10 mM HEPES + P/S	5
A549	Lung adenocarcinoma	DMEM + 10% FCS + P/S	7.5
CALU6	Lung adenocarcinoma	MEM (+ Earle's salts), 10% FCS + P/S	5
COLO699	Lung adenocarcinoma	RPMI-1640 + 10% FCS + 10 mM HEPES + P/S	5
ETCC016	Lung adenocarcinoma	DMEM + 10% FCS + P/S	7.5
HCC44	Lung adenocarcinoma	RPMI-1640 + 10% FCS + 10 mM HEPES + P/S	5
HCC78	Lung adenocarcinoma	RPMI-1640 + 10% FCS + 10 mM HEPES + P/S	5
HCC827	Lung adenocarcinoma	RPMI-1640 + 15% FCS + 10 mM HEPES + P/S	5
LXF289	Lung adenocarcinoma	Ham's F10 + 10% FCS + P/S	5
SKLU1	Lung adenocarcinoma	EMEM + 10% FCS, + P/S	5
A172	Glioblastoma	DMEM + 10% FCS + P/S	7.5
LN18	Glioblastoma	DMEM + 10% FCS + P/S	7.5
LN229	Glioblastoma	DMEM + 10% FCS + P/S	7.5
T98G	Glioblastoma	DMEM + 10% FCS + P/S	7.5
U87	Glioblastoma	DMEM + 10% FCS + P/S	7.5
U138	Glioblastoma	DMEM + 10% FCS + P/S	7.5
U251	Glioblastoma	DMEM + 10% FCS + P/S	7.5
Cal27	Head and neck cancer	DMEM + 10% FCS + P/S	7.5
Cal33	Head and neck cancer	DMEM + 10% FCS + P/S	7.5
UDSCC2	Head and neck cancer	DMEM + 10% FCS + P/S	7.5
UMSCC1	Head and neck cancer	DMEM + 10% FCS + P/S	7.5

UPCISCC040	Head and neck cancer	DMEM + 10% FCS + P/S	7.5
UPCISCC099	Head and neck cancer	DMEM + 10% FCS + P/S	7.5
UPCISCC131	Head and neck cancer	DMEM + 10% FCS + P/S	7.5
UPCISCC154	Head and neck cancer	DMEM + 10% FCS + P/S	7.5
UTSCC16A	Head and neck cancer	DMEM + 10% FCS + P/S	7.5
93-VU-147T	Head and neck cancer	DMEM + 10% FCS + P/S	7.5
BT20	Breast cancer	DMEM/F12 (1 + 1) + 10% FCS + P/S	7.5
BT474	Breast cancer	RPMI-1640 + 10% FCS + 10 mM HEPES + P/S	5
BT549	Breast cancer	DMEM + 10% FCS + P/S	7.5
EFM19	Breast cancer	RPMI-1640 + 10% FCS + 10 mM HEPES + P/S	5
HCC1806	Breast cancer	RPMI-1640 + 10% FCS + 10 mM HEPES + P/S	5
HCC1937	Breast cancer	RPMI-1640 + 10% FCS + 10 mM HEPES + P/S	5
MCF7	Breast cancer	RPMI-1640 + 10% FCS + 10 mM HEPES + P/S	5
MDA-MB231	Breast cancer	DMEM + 10% FCS + P/S	7.5
MDA-MB436	Breast cancer	DMEM/F12 (1 + 1) + 10% FCS + P/S	7.5
MDA-MB453	Breast cancer	DMEM + 10% FCS + P/S	7.5
MDA-MB468	Breast cancer	DMEM/F12 (1 + 1) + 10% FCS + P/S	7.5
SKBR3	Breast cancer	DMEM + 10% FCS + P/S	7.5
T47D	Breast cancer	RPMI-1640 + 10% FCS + 10 mM HEPES + P/S	5
ZR75-1	Breast cancer	RPMI-1640 + 10% FCS + 10 mM HEPES + P/S	5

Supplementary Table S2: Overview on cell lines and culture media used in this study. FCS (fetal calf serum), P/S (100 U/ml penicillin and 0.1 mg/ml streptomycin).
